# Supplementary material for: Single-molecule FRET uncovers hidden conformations and dynamics of human Argonaute 2
Source: Nat Commun. 2022 Jul 2;13:3825. doi: 10.1038/s41467-022-31480-4 (PMC9250533; doi:10.1038/s41467-022-31480-4)
Supplement: Supplementary file 1 — Supplementary Information [file 41467_2022_31480_MOESM1_ESM.pdf]

Supplementary Figures

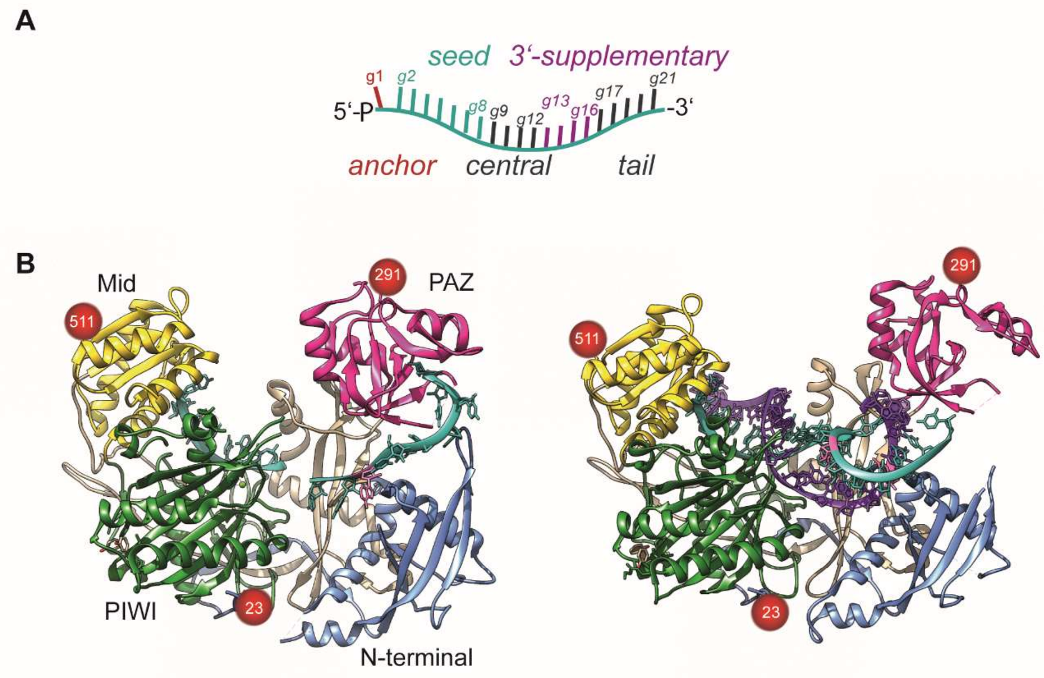

	guide 3'-end PAZ attached			guide 3'-end released	
	binary pdb 4W5N	seed-match pdb 4W5T	seed + suppl. match pdb 6N4O	10-11 mm pdb 6MDZ	9-12 mm pdb 6NIT
291 - g14	4.1 nm	4.5 nm	4.0 nm	4.7 nm	4.6 nm
511 - g14	6.8 nm	5.9 nm	5.6 nm	5.5 nm	5.5 nm
291 - 23	6.6 nm	7.4 nm	7.7 nm	7.9 nm	7.8 nm
511 - 23	6.3 nm	6.4 nm	6.5 nm	6.3 nm	6.2 nm
291 - 511	6.2 nm	6.9 nm	7.3 nm	7.8 nm	7.7 nm
291 - t4			4.1 nm	4.4 nm	4.4 nm

Figure S1 (related to Figure 1): Labeling positions in hAgo2 used in this study, distances between labels derived from crystal structures and functional domains of an Ago-bound guide. (A) Functional domains of Ago-bound guide RNA. Figure modified from Wee et al. [1]**.** (B) Domains of hAgo2 are defined according to Elkayam et al. (2012) [2]. The pdb identifiers of the crystal structures of hAgo2 are 4W5N (left) and 6MDZ (right) [3,4]. To determine theoretical distances between labels, the amino acids at the labeling positions in the respective structures were replaced by phenylalanines to mimic the residue p-azido-L-phenylalanine used in this study. The labeling positions in guide and target RNA were exchanged by an uracil to mimic the nucleotides in the guide and target strands used in this study. The distances specified in the table were determined based on the crystal structures listed. Distances were measured between para-C atoms of the phenylalanines or between the para-C atom of phenylalanine and atom C5 of the uracil base in labeling positions of the RNA.

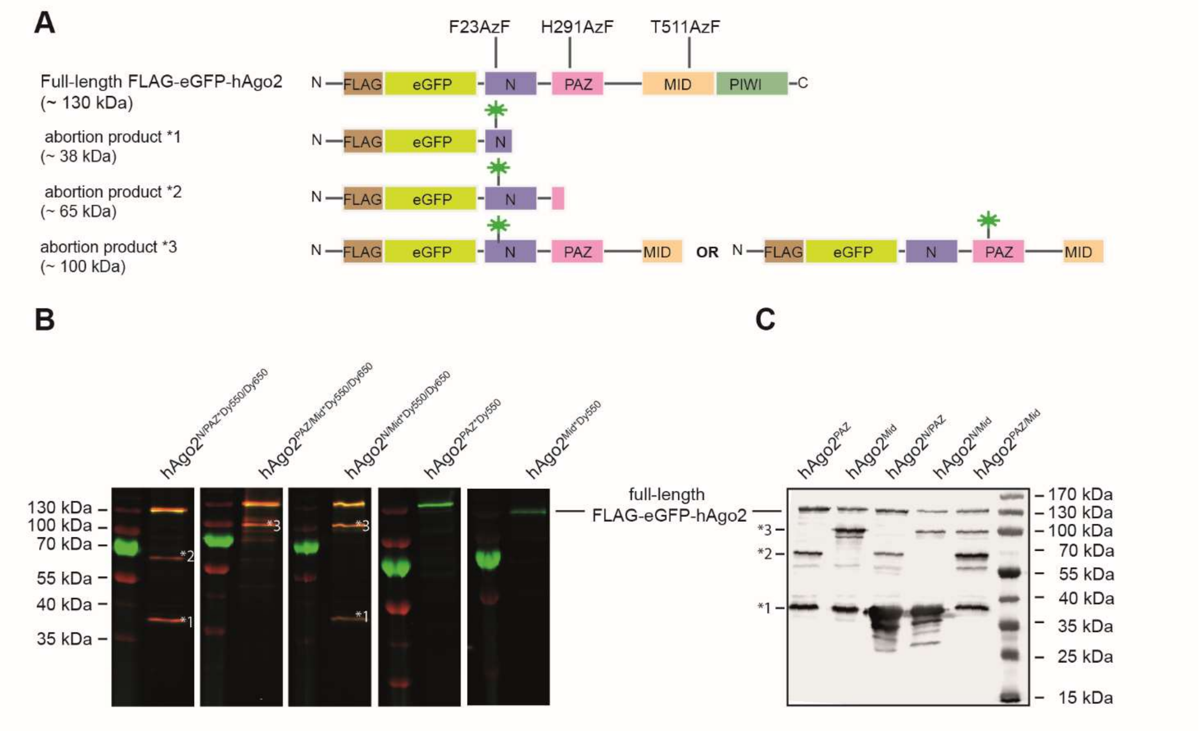

Figure S2 (related to Figure 1): Site-specific engineering of an unnatural amino acid into FLAG-eGFP-hAgo2 followed by fluorescent labelling. (A) Variants of full-length FLAG-eGFP-hAgo2 used in this the study. Residues F23, H291 and T511 were replaced by the unnatural amino acid p-Azidophenylalanine (AzF). Fluorescently labeled abortion products that occur during translation are depicted. (B) Fluorescence scans of SDS PAGE analysis of either DyLight™ 550-labeled single mutants or double mutants stochastically labeled with DyLight™ 550 and DyLight™ 650. (C) Western blot analysis of hAgo2 variants using a primary antibody directed against the N-terminus of hAgo2 (primary antibody 11A9, [5]). Representative result from two biological replicates is shown.

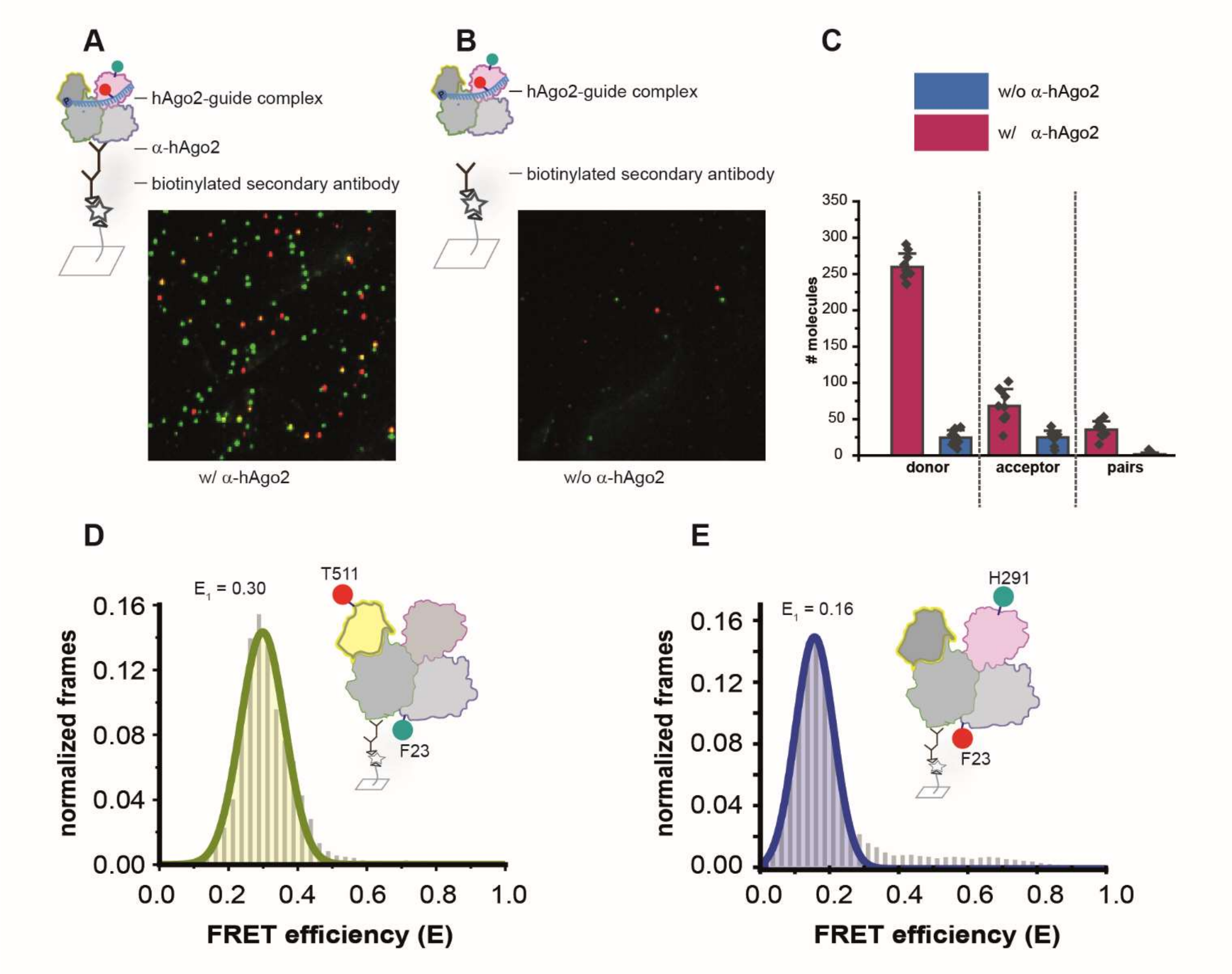

Figure S3 (related to Figure 1): Immobilization of labeled hAgo2 using hAgo2-directed antibodies. (A) and (B) show EMCCD camera images of measurements of binary complexes (hAgo2^{PAZ}-guide RNA^{14Cy5}) immobilized using a biotinylated antibody directed against a hAgo2-specific antibody (α-hAgo2, primary antibody 11A9, [5]) with an overlay of donor (green signal) and acceptor (red signal) channel. (B) shows a sample in which the hAgo2-specific antibody was omitted. (C) Quantitative evaluation of donor, acceptor and donor/acceptor molecules recorded with or without the hAgo2-specific antibody using hAgo2^{PAZ}-guide RNA^{14Cy5} complexes. Bars represent the mean of the evaluation of 10 videos and the error bars represent the corresponding standard deviations. (D) and (E) show FRET efficiency histograms of measurements conducted with an hAgo2 antibody directed against the PIWI domain (abcam #ab57113) instead of the N-terminal domain. The FRET efficiency histogram in (D) for the hAgo2$^{N/Mid}$ apo enzyme shows the mean of two independent measurements fitted by a single Gaussian equation, whereas the histogram shown in (E) is derived from smFRET measurements with hAgo2$^{N/PAZ}$ apo enzyme and shows the mean of three independent measurements fitted by a single Gaussian fit. Source data provided as Source Data file.

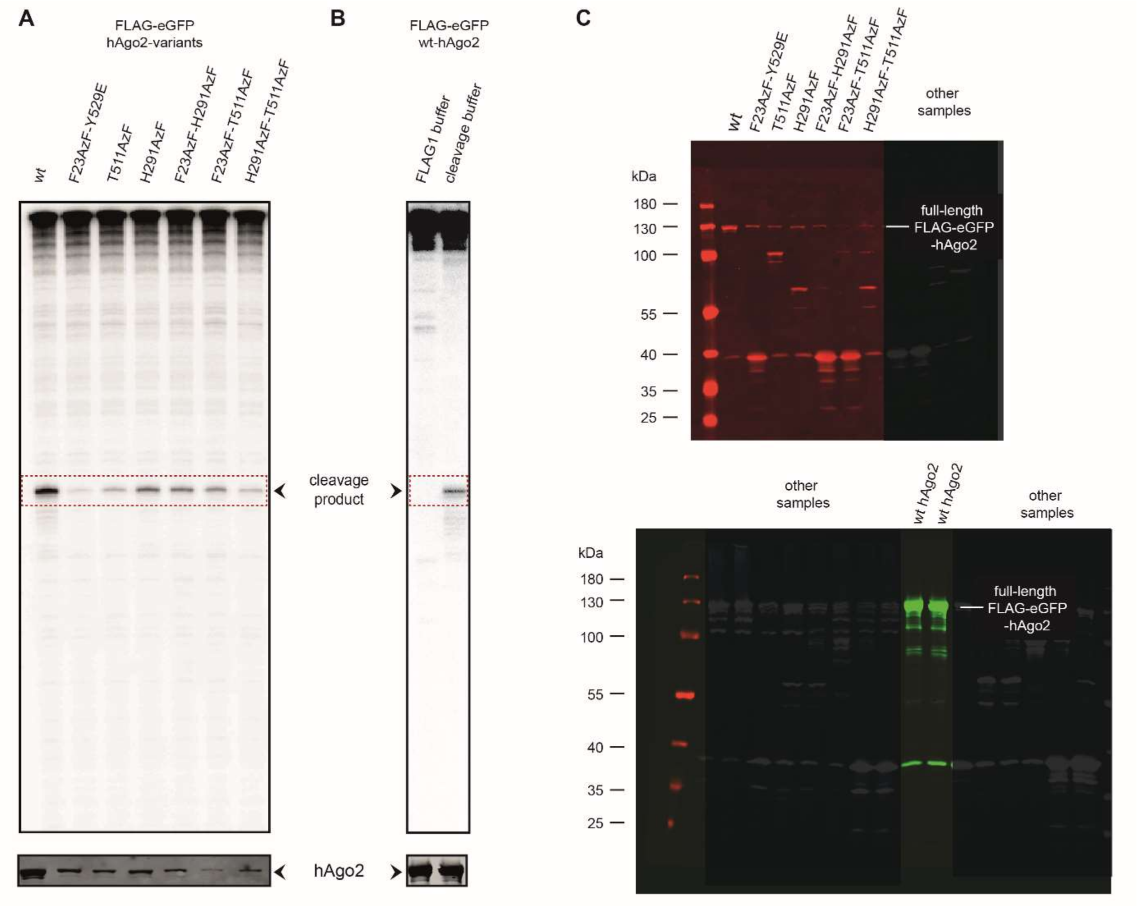

Figure S4 (related to Figure 1): *In vitro* cleavage activity of immunopurified FLAG-eGFP-hAgo2-variants expressed in HEK293 T. (A) *In vitro* cleavage assay of hAgo2 variants using radio-labeled target RNA. After immunoprecipitation a radioactively labeled substrate was added. The substrate is fully complementary to the endogenous miR-19b, which is present in a fraction of immunopurified hAgo2 and its variants. The experiment was conducted three times independently and a representative gel is shown. For control, a part of the beads was used for Western blotting (lower panel). (B) The FLAG I buffer, which is used for the TIRF measurements, allows for nucleic acid binding but prohibits cleavage. (C) Uncropped images of the Western Blots shown in the lower panels of (A: upper Blot) and (B: lower Blot). Other samples are samples that are not relevant for the data shown here and are covered by a transparent black box.

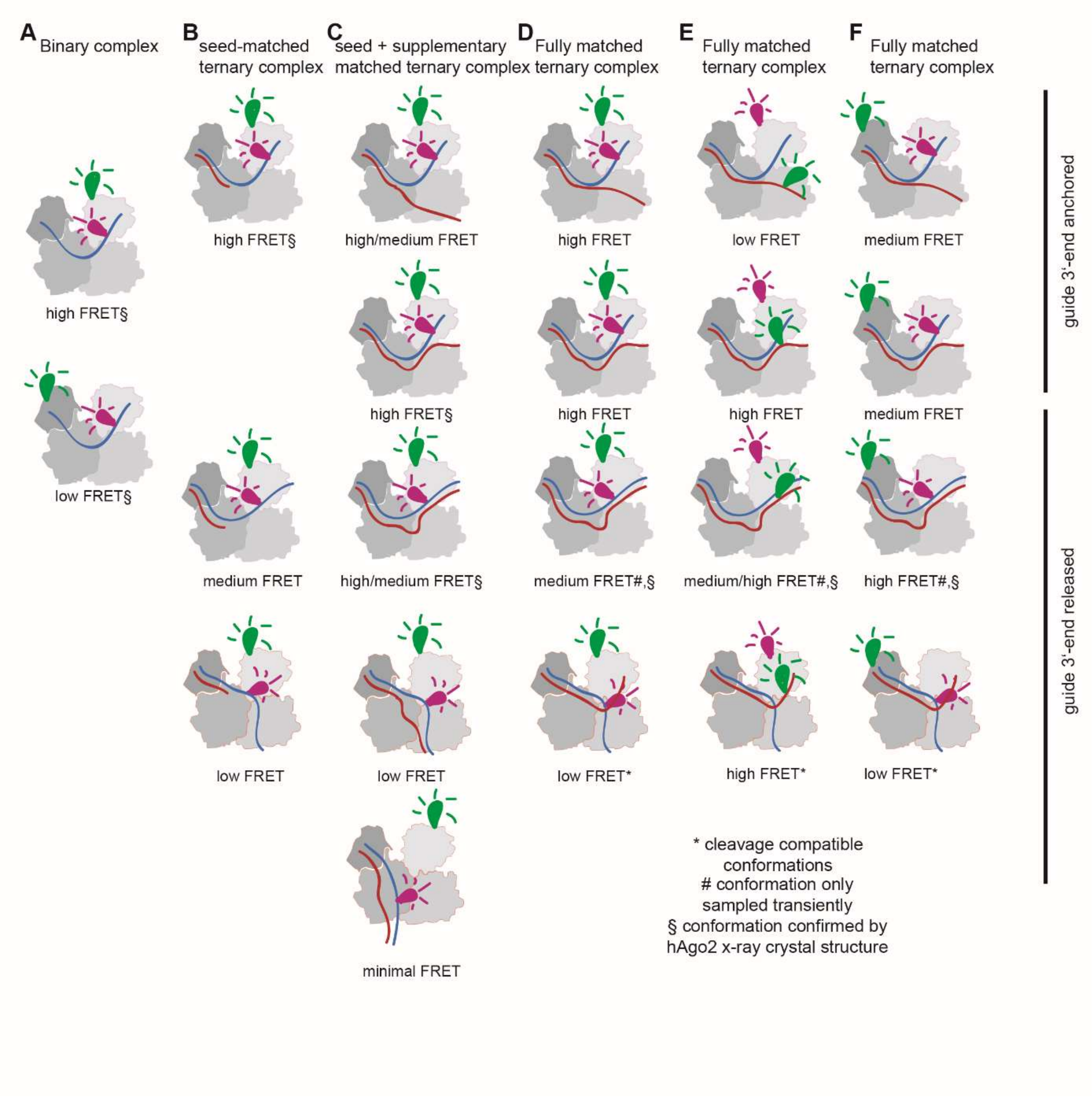

Figure S5 (related to Figure 2): Schematic illustration of complex conformations assigned to the FRET populations observed in smFRET measurements. This figure depicts guide and target trajectories within binary and ternary complexes that we suggest based on the results of our smFRET measurements. Schematic representations are based on the following crystal structures: pdb identifiers: 4W5N, 4W5T, 6MDZ, 6NIT, 6N4O.

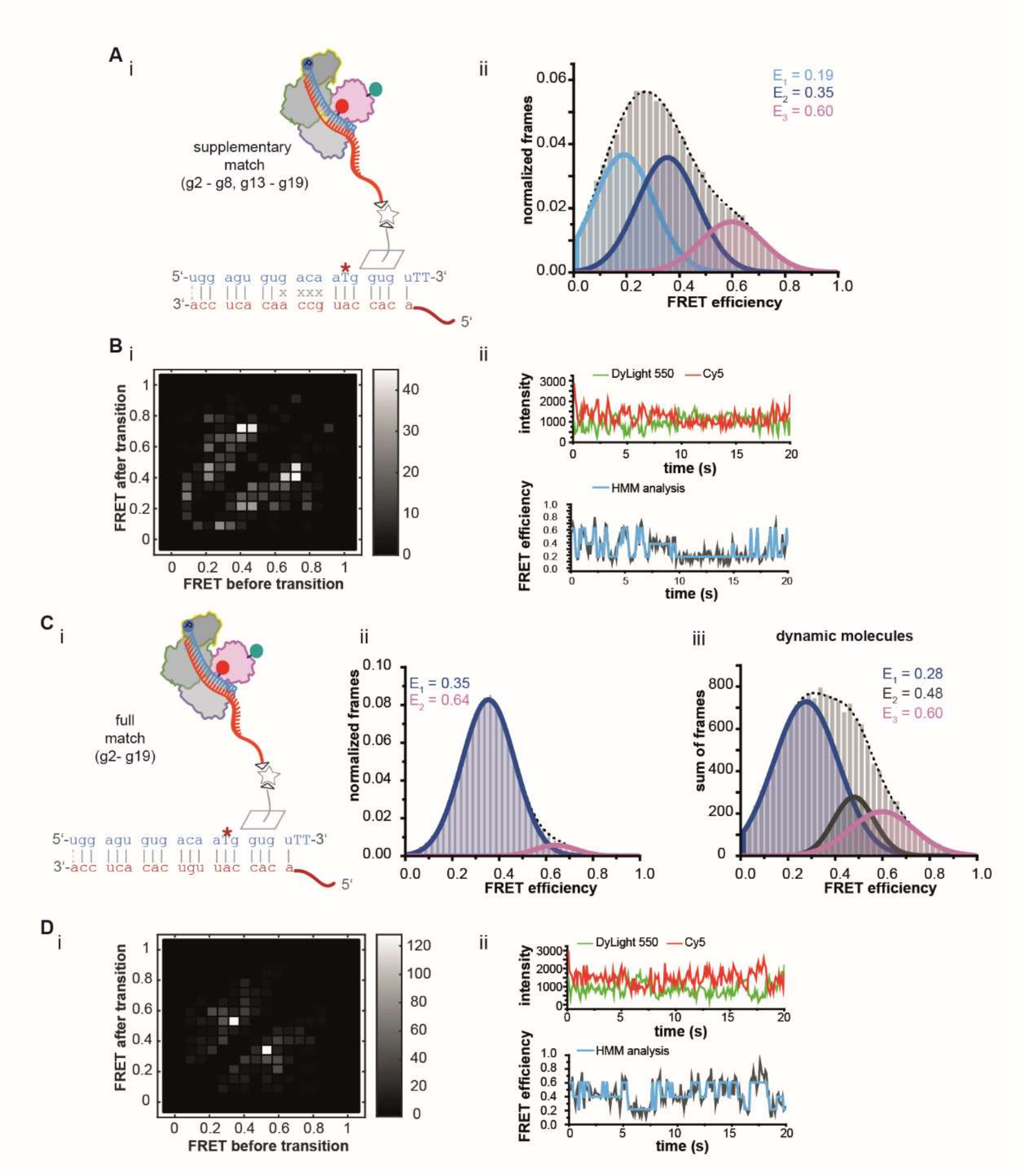

Figure S6 (related to Figure 2): Conformational states and dynamic behavior of ternary hAgo2-guide/target complexes with an alternative guide/target pair. (A) Measurement of ternary complexes composed of hAgo2^{PAZ}, labeled guide miRNA122^{14Cy5} and target miRNA122$^{biotin\text{-}mm9\text{-}12}$. Complexes were immobilized using the biotin modification at the 5'-end of the target RNA (i). The FRET efficiency histogram shows data of five independent measurements. The FRET efficiency distribution was analyzed using a trimodal Gaussian distribution (ii). (B) Analysis of all dynamic molecules detected in the measurements presented in (A, ii). Transitions between FRET states are presented in a transition density plot. (i) Representative fluorescence transients with corresponding FRET efficiency trace fitted with Hidden-Markov-Model analysis (HMM) for the complexes depicted in (A, ii). (C) Measurement of ternary complexes composed of hAgo2^{PAZ}, labeled guide miRNA122^{14Cy5} and target miRNA122$^{biotin\text{-}fullmatch}$. Complexes were immobilized using the biotin modification at the 5'-end of the target RNA (i). The FRET efficiency histogram shows data of three independent measurements and is analyzed using a double Gaussian distribution (ii) Analysis of dynamic molecules in case of the full matched target RNA reveals the presence of a hidden third conformation with a medium FRET efficiency of E = 0.48 (iii). (D)

Analysis of all dynamic molecules detected in the measurements presented in (C, ii, iii). Transitions between FRET states are presented in a transition density plot (i). Representative fluorescence transients with corresponding FRET efficiency trace fitted with Hidden-Markov-Model analysis (HMM) for the complexes depicted in (C, i).

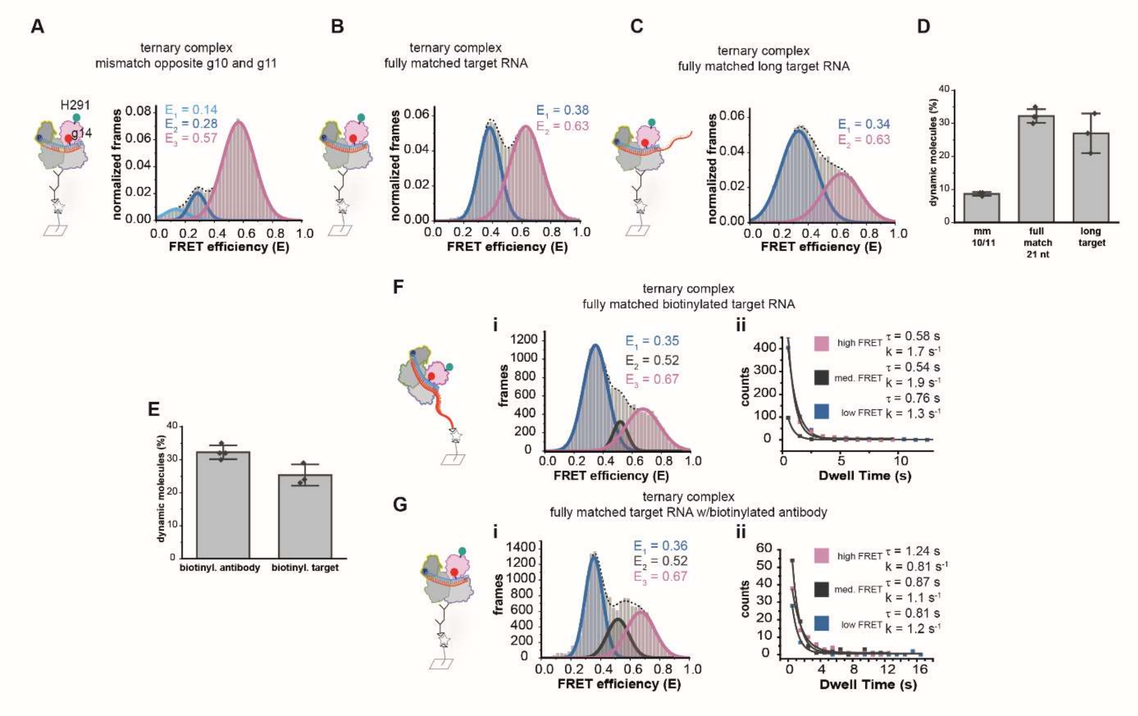

Figure S7 (related to Figure 2): Conformational states and dynamic behavior of ternary hAgo2-guide/target complexes. (A) Measurement of ternary complexes composed of $hAgo2^{PAZ}$, labeled guide RNA^{14Cy5} and target $RNA^{mm10/11}$. The FRET efficiency histogram represents the mean of three independent measurements and is analyzed using a triple Gaussian. B) Measurement of ternary complexes composed of $hAgo2^{PAZ}$, labeled guide RNA^{14Cy5} and target RNA^{21nt}. Complexes were immobilized using the hAgo2-directed antibody. The FRET efficiency histogram represents the mean of four independent measurements and is analyzed using a double Gaussian (mean FRET efficiencies are listed in the panel). (C) Measurement of ternary complexes composed of $hAgo2^{PAZ}$, labeled guide RNA^{14Cy5} and target RNA^{long}. Complexes were immobilized using the hAgo2-directed antibody. The FRET efficiency histogram represents the mean of three independent measurements and is analyzed using a double Gaussian. Mean FRET efficiencies are indicated in the panel. (D) Fraction of dynamic molecules detected in the respective measurements. Bars show the mean of at least three independent measurements and the error bars represent standard deviations (no. of biological replicates: mm 10/11 = 3; full match 21 nt = 4; long target = 3). (E) Fraction of dynamic molecules as determined in measurements of hAgo2 in complex with fully matched target RNAs. Bars show the mean of at least three independent measurements. Error bars represent standard deviations (no. of biological replicates: biotinyl. antibody = 4 (full match 21 nt S3D); biotinyl. target = 3 (full match 2E)). (F) and (G) (i) FRET efficiency histograms reflecting the FRET distribution of molecules that showed dynamic FRET time traces in the respective measurements (ii) Analysis of dwell times (τ) and the resulting rate constants (*k*) deduced from the analysis of dynamic time traces of all dynamic molecules

in the respective measurements. (F) Ternary complexes immobilized via the target RNA$^{long\text{-}Biotin}$. (G) Ternary complexes immobilized via a biotinylated antibody. Source data provided as a Source Data file.

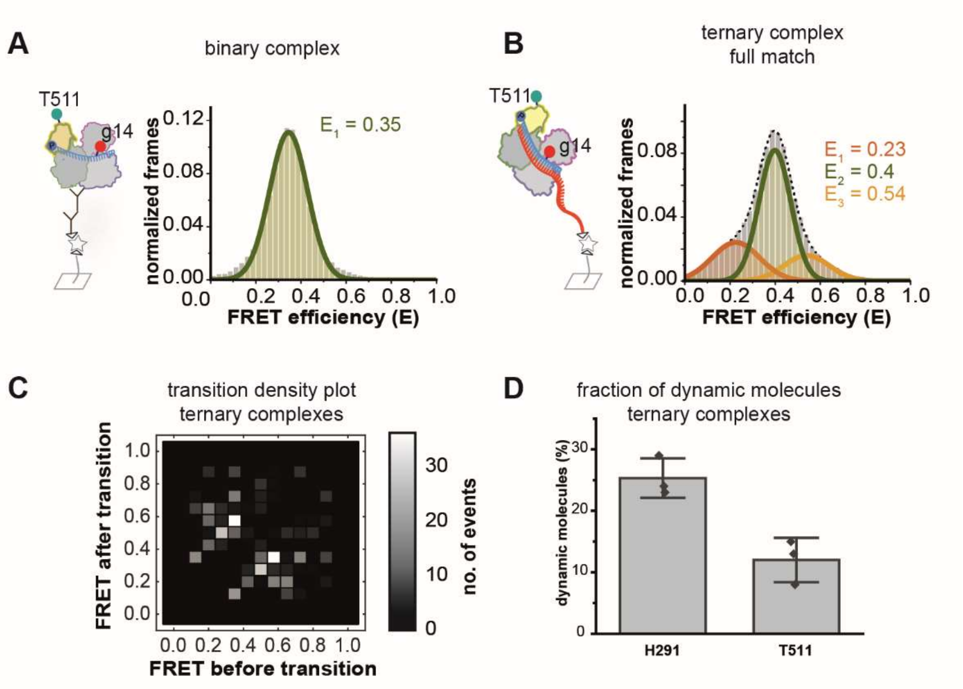

Figure S8 (related to Figure 2): Single-molecule FRET analysis of binary and ternary hAgo2 complexes carrying a donor in the Mid domain and an acceptor dye positioned in the guide strand. Measurement of (A) binary complexes composed of hAgo2^{Mid*DL550} and guide RNA^{14Cy5} and (B) ternary complexes composed of hAgo2^{Mid*DL550}, guide RNA^{14Cy5} and target RNA$^{long\text{-}Biotin}$. The FRET efficiency histograms represent the mean of three independent measurements. Data were analyzed using a triple Gaussian equation. Mean FRET efficiencies are given in the panels. (C) Analysis of all dynamic molecules detected in the measurements presented in (B). Transitions between FRET states are presented in a transition density plot. (D) Comparison of the fraction of dynamic molecules detected in measurements of ternary complexes using either a hAgo2 variant labeled with a donor fluorophore in the PAZ (hAgo2^{PAZ}) or Mid domain (hAgo2^{Mid}). Bars represent the mean of three independent measurements and error bars show the corresponding standard deviation (ternary complexes with hAgo2^{PAZ} compare Fig. 2E and S7E). Source data provided as a Source Data file.

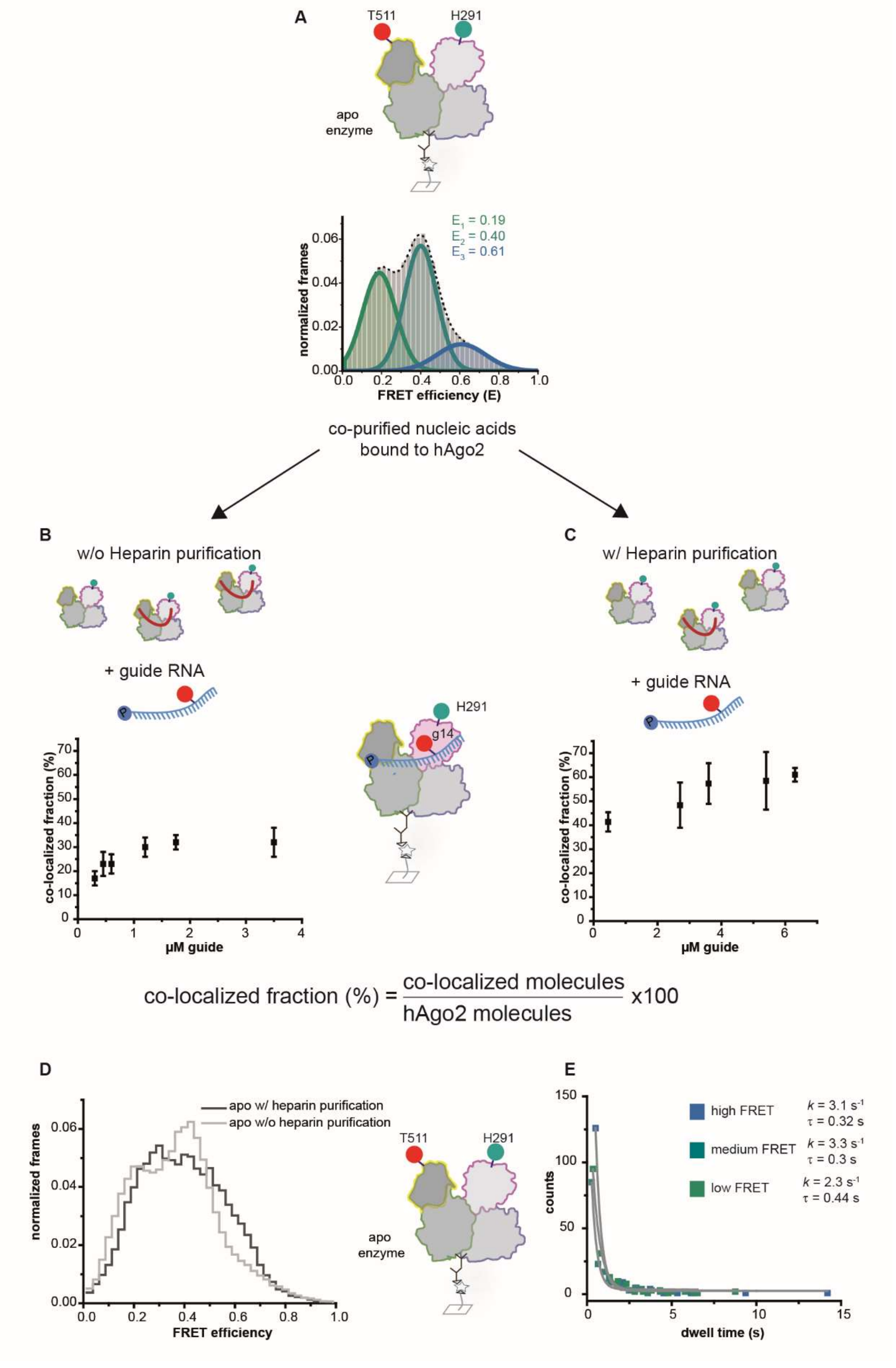

Figure S9 (related to Figure 3): Analysis of nucleic acid free hAgo2 molecules versus hAgo2 molecules with co-purified nucleic acids. (A) Doubly labeled apo hAgo2$^{PAZ/Mid}$ was immobilized via an hAgo2-directed antibody. The FRET efficiency histogram shows data from four independent measurements. Data were fitted using a triple Gaussian equation. As hAgo2 was not purified via a heparin affinity column, hAgo2 molecules expressed and purified from HEK cells contain a fraction of molecules with

co-purified nucleic acids. To determine the fraction of apo hAgo2 in the sample, single-labeled hAgo2^{PAZ} was incubated with increasing amounts of labeled guide^{RNA14Cy5}. Guide concentrations used for complex formation with 1/6th of hAgo2^{PAZ} purified from cell pellets derived from two wells of a 6-well plate (B) or 1/5th of purified hAgo2 from the selected peak fraction (C) are shown on the respective x-axes. Molecules that bind labeled guide RNA are considered the fraction of apo hAgo2 that can be loaded with synthetic RNA. (B) Data points represent the mean of x independent measurements (w/o heparin purification: 0.45 µM, 3.5 µM: x = 3; 0.3 µM, 0.6 µM, 1.75 µM: x = 4, 1.2 µM: x = 5; w/ heparin purification: 0.45 µM, 2.7 µM, 3.6 µM: x = 3; 5.4 µM, 6.3 µM: x = 2) with the error bars displaying the standard deviation. Source data provided as a Source Data file. (C) In order to prepare hAgo2 in a nucleic acid free state, an additional purification step using a Heparin affinity column was performed before the titration experiment. Data points represent the mean of at least two measurements with the error bars displaying the standard deviation. (D) Comparison of FRET efficiency histograms from measurements of apo hAgo2$^{PAZ/Mid}$ with and without additional purification employing Heparin affinity chromatography. (E) Dwell time analysis of the apo enzyme analyzed using a single exponential decay function. Dwell times (τ) and rate constants (k) are shown in the graph.

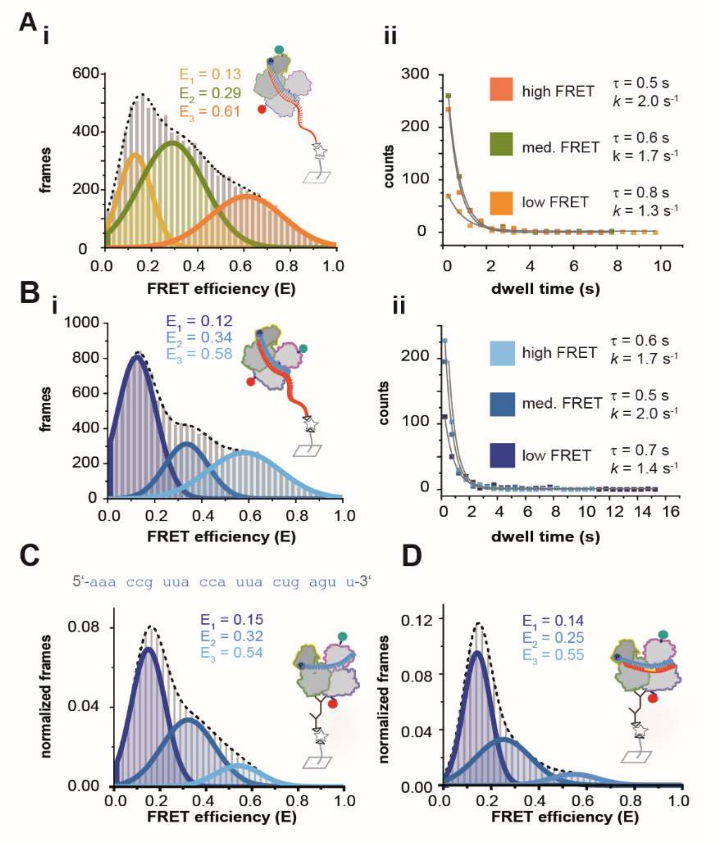

Figure S10 (related to Figures 4 and 5): Intramolecular FRET reports on the dynamics of hAgo2's C and N-terminal lobe and on conformational changes upon binding of guide and mismatched target RNA. (A) (i) FRET efficiency histogram based on FRET values detected for dynamic molecules in measurements of ternary hAgo2$^{N/Mid}$-guide RNA-target RNA$^{long\text{-}Biotin}$ complexes. The FRET efficiency histogram shows the sum of all frames of dynamic molecules taken from four independent measurements. Data was analyzed using a triple Gaussian function. FRET efficiencies are indicated in the graph. (ii) Data in the resulting dwell time histograms were analyzed using a single exponential decay function. Dwell times (τ) and rate constants (k) are shown in the graph. (B) (i) FRET efficiency histogram based on FRET values detected for dynamic molecules in measurements of ternary hAgo2$^{N/PAZ}$-guide RNA-target RNA$^{long\text{-}Biotin}$ complexes. The FRET efficiency histogram shows the sum of all frames of the dynamic molecules only. Data were analyzed using a triple Gaussian function. FRET efficiencies are listed in the graph. (ii) Data in the resulting dwell time histograms were fit using a single exponential decay function. Dwell times (τ) and rate constants (k) are shown in the graph. (C) SmFRET measurements of hAgo2 labeled in the N-terminal and PAZ domain in complex with an unlabeled miRNA guide. The FRET efficiency histogram shows mean normalized frames of three independent measurements. Data were fitted using a triple Gaussian function and mean FRET efficiencies are indicated in the panel. (D) SmFRET measurements of hAgo2 labeled in the N-terminal and PAZ domain in complex with an unlabeled guide RNA bound to target RNA$^{21nt\text{-}mm9\text{-}12}$. The FRET efficiency histogram shows mean normalized frames of four independent measurements. Data were analyzed using a triple Gaussian function and mean FRET efficiencies are indicated in the panel. Source data provided as a Source Data file.

Supplementary Tables

Supplementary Table 1: Sequences of RNA nucleotides used in the present study

NAME	SEQUENCE
guide RNA	5’-phosphate- uag agg uac gug cug agg cTT-3’
guide RNA^{14Cy5}	5‘-phosphate- uag agg uac gug cXg agg cTT-3‘ *X = U-Cy5*
target RNA^{21nt}	5’-gcc uca gca cgu acc ucu aTT-3’
target RNA$^{4\text{-}Cy3}$	5’-gcc Xca gca cgu acc ucu aTT-3’ *X=T-Cy3*
target RNA$^{mm10/11}$	5'-gcc uca gcc agu acc ucu aTT-3'
target RNA^{seed}	5’-aaa cac aac acu acc ucu aTT-3’
target RNA$^{long\text{-}Biotin}$	5'-biotin-ccg cag uca uaa ugg gca cug cag gcc uca gca cgu acc ucu a-3'
target RNA^{long}	5'-ccg cag uca uaa ugg gca cug cag gcc uca gca cgu acc ucu a-3'
target RNA$^{longmm9\text{-}12\text{-}Biotin}$	5'-biotin-ccg cag uca uaa ugg gca cug cag gcc uca ggc acu acc ucu a-3'
pre-miR451a	5'-aaa ccg uua cca uua cug agu uua gua aug gua acg guu cu-3'
guide RNA$^{p\text{-}miR451a}$	5'-phosphate-aaa ccg uua cca uua cug agu u-3'
target RNA^{osr1}	5'-aac uau uaa aua aac ggu uau u-3'
target RNA$^{21nt\text{-}mm9\text{-}12}$	5’-gcc uca ggc acu acc ucu aTT-3’
guide miRNA122^{14Cy5}	5’-phosphate-ugg agu gug aca aXg gug u TT-3’ *X = T-Cy5*
target miRNA122$^{biotin\text{-}fullmatch}$	5’-biotin-ccg cag uca uaa ugg gca cug cag aca cca uug uca cac ucc a-3’
target miRNA122$^{biotin\text{-}mm9\text{-}12}$	5’-biotin-ccg cag uca uaa ugg gca cug cag aca cca ugc caa cac ucc a-3’
target RNA cleavage assay	5’-gaa caa uug cuu uua cag aug cac aua ucg agg uga aca uca cgu acu cag uuu ugc aug gau uug cac auc ggu ugg cag aag cua uga aac gau aug ggc uga aua caa auc aca gaa ucg ucg uau gca gug aaa acu cuc uuc aau ucu uua ugc cua uag ugu cac cua aau-3’

**all oligonucleotides with the identifier ‘Biotin’ carry a Biotin tag at their 5’-end.*

Supplementary Table 2: Summary of all SmFRET measurements

Black lines represent the mean of all measurements and grey areas represent standard deviations. *width of population fixed.

Nucleic acid binding partner	Immob. method	E1	E2	E3	R^2	No. of molecules	No. of replicates	mean FRET efficiency & standard deviation
**hAgo2^{PAZ*DyLight550}**								
guide RNA^{14Cy5}	11A9	0.64 +/- 0.002	-	-	0.98	432	3	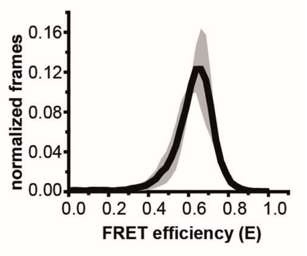
guide RNA^{14Cy5} target RNA^{seed}	11A9	0.34 +/- 0.03	0.47 +/- 0.008	0.62 +/- 0.008	0.99	179	4	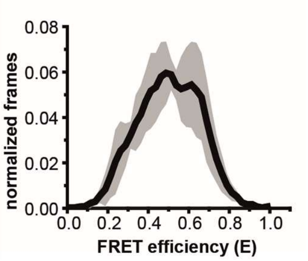
guide RNA^{14Cy5} target RNA^{21nt}	11A9	0.38 +/- 0.002	0.63 +/- 0.002	-	0.99	361	4	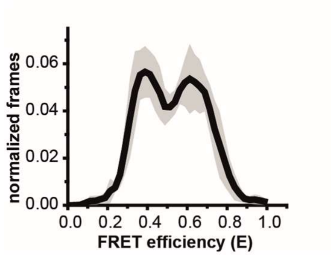
guide RNA^{14Cy5} target RNA$^{long-Biotin}$	Biotin target	0.35 +/- 0.002	0.59 +/- 0.01	-	0.99	487	3	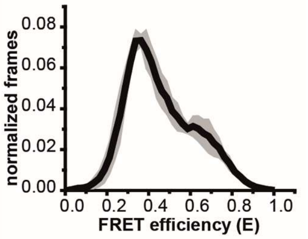
guide RNA^{14Cy5} target RNA^{long}	Biotin target	0.34 +/- 0.006	0.63 +/- 0.01	-	0.99	190	3	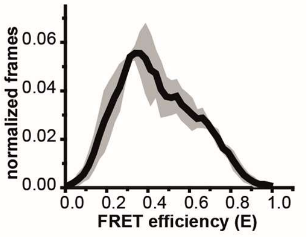

**hAgo2^{PAZ*DyLight550}**								
guide RNA^{14Cy5} target RNA$^{longmm9-12-Biotin}$	Biotin target	0.16 +/- 0.01	0.34 +/- 0.01	0.54 +/- 0.01	0.99	302	4	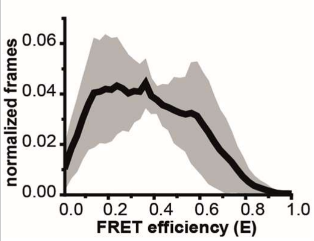
guide RNA^{14Cy5} target RNA$^{mm10/11}$	11A9	0.14 +/- 0.03	0.28 +/- 0.009	0.57 +/- 0.001	0.99	308	3	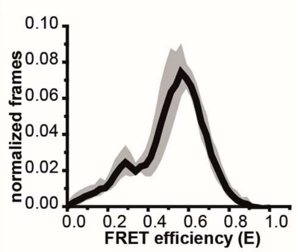
guide miRNA122^{14Cy5} target miRNA122$^{Biotin-mm9-12}$	Biotin target	0.19 +/- 0.03	0.35* +/- 0.04	0.60 +/- 0.02	0.99	455	5	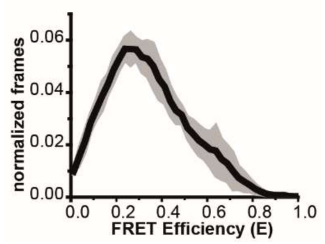
guide miRNA122^{14Cy5} target miRNA122$^{Biotin-fullmatch}$	Biotin target	0.35 +/- 0.002	0.64 +/- 0.02	-	0.99	442	3	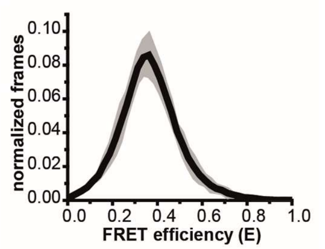
**hAgo2^{PAZ*DyLight650}**								
guide RNA target RNA^{4-Cy3}	11A9	0.32 +/- 0.01	0.59 +/- 0.006	-	0.99	165	3	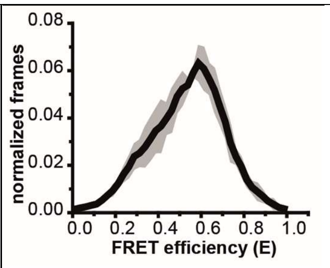

**hAgo2^{Mid*DyLight550}**								
guide RNA^{14Cy5}	11A9	0.34 +/- 0.001	-	-	0.99	391	4	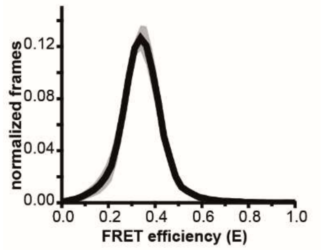
guide RNA^{14Cy5} target RNA$^{long-Biotin}$	Biotin target	0.23 +/- 0.02	0.40 +/- 0.002	0.54* +/- 0.01	0.99	352	3	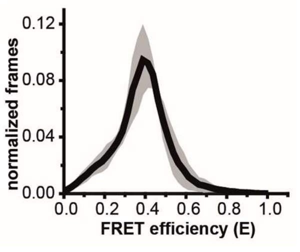
**hAgo2$^{PAZ/Mid*DyLight550/DyLight650}$**								
apo	11A9	0.19 +/- 0.002	0.40 +/- 0.002	0.61* +/- 0.007	0.99	681	4	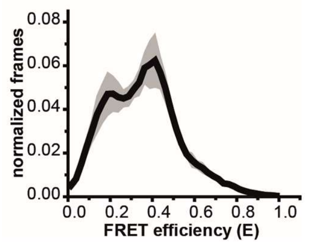
apo (modified purification protocol)	11A9	0.24 +/- 0.01	0.42 +/- 0.02	0.53 +/- 0.07	0.99	206	4	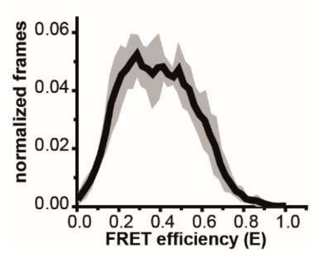
guide RNA (modified purification protocol)	11A9	0.18 +/- 0.009	0.36 +/- 0.02	0.59* +/- 0.09	0.99	181	3	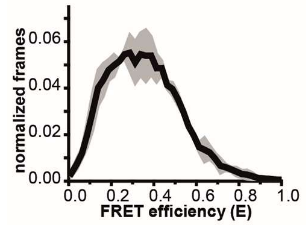
guide RNA target RNA$^{long-Biotin}$	Biotin target	0.19 +/- 0.01	0.37 +/- 0.01	-	0.99	577	3	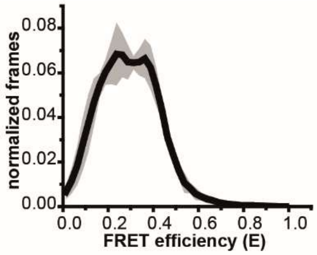

**hAgo2$^{N/Mid*DyLight550/DyLight650}$**								
apo	11A9	0.28 +/- 0.002	-	-	0.98	1175	5	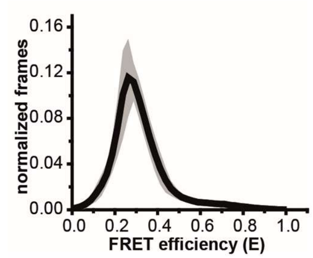
guide RNA	11A9	0.1 +/- 0.008	0.3* +/- 0.003	0.58 +/- 0.02	0.99	531	3	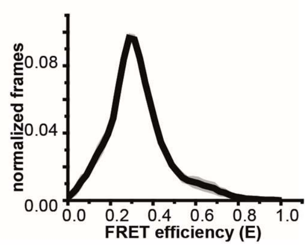
guide RNA target RNA$^{long-Biotin}$	Biotin target	0.07 +/- 0.01	0.29* +/- 0.003	0.56 +/- 0.02	0.99	702	4	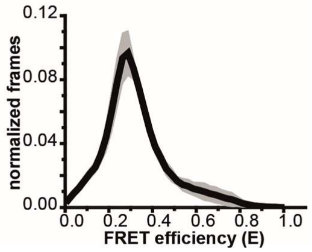
apo	ab57113	0.30 +/- 0.002	-	-	0.97	180	2	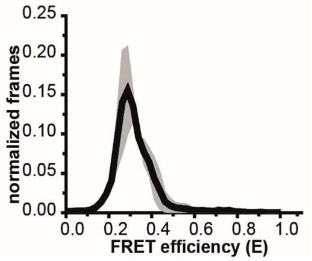
**hAgo2$^{N/PAZ*DyLight550/DyLight650}$**								
apo	11A9	0.16 +/- 0.003	-	-	0.97	696	3	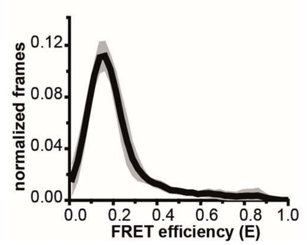
apo	ab57113	0.16 +/- 0.002	-	-	0.97	325	3	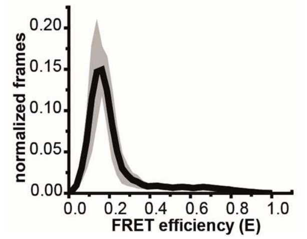

**hAgo2$^{N/PAZ*DyLight550/DyLight650}$**								
guide RNA	11A9	0.18* +/- 0.002	0.36 +/- 0.009	0.51 +/- 0.09	0.99	611	3	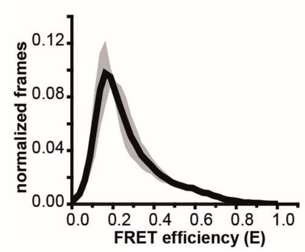
guide RNA target RNA$^{long-Biotin}$	Biotin target	0.14* +/- 0.003	0.36 +/- 0.02	0.53 +/- 0.09	0.98	772	4	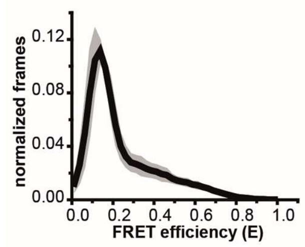
pre-miR451a	11A9	0.16 +/- 0.0001	0.31 +/- 0.03	-	0.99	535	4	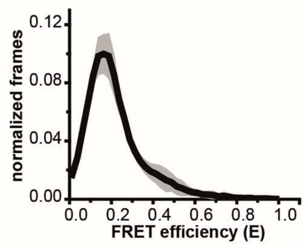
guide RNA$^{p-miR451a}$	11A9	0.15 +/- 0.001	0.32* +/- 0.01	0.54* +/- 0.01	0.99	346	3	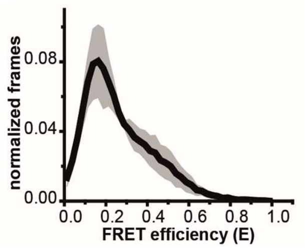
guide RNA$^{p-miR451a}$ target RNA^{osr1}	11A9	0.16 +/- 0.001	0.31 +/- 0.03	-	0.99	472	4	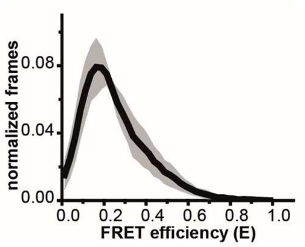

Supplementary Table 3: Experimental settings used for different donor-acceptor fluorophore combinations.

Fluorophores (donor | acceptor)	Excitation wavelength and power	Emission filters
Cy3 | DyLight650	532 nm, 30 mW	HC BS 640 (Semrock)

	637 nm, 50 mW	582/75 Brightline HC (Semrock)<br>635 LP Edge Basic (Semrock)
Dylight550 | Dylight650 or<br>Dylight550 | Cy5	561 nm, 20 mW<br>637 nm, 30 mW	HC BS 640 (Semrock)<br>595/50 H Bandpass (Semrock)<br>635 LP Edge Basic (Semrock)

Supplementary References

1. Wee LM, Flores-Jasso CF, Salomon WE, Zamore PD. Argonaute divides its RNA guide into domains with distinct functions and RNA-binding properties. *Cell*. 2012;151(5):1055-1067. doi:10.1016/j.cell.2012.10.036
2. Elkayam E, Kuhn C-DD, Tocilj A, et al. The structure of human argonaute-2 in complex with miR-20a. *Cell*. 2012;150(1):100-110. doi:10.1016/j.cell.2012.05.017
3. Sheu-Gruttadauria J, Pawlica P, Klum SM, et al. Structural Basis for Target-Directed MicroRNA Degradation. *Mol Cell*. 2019;75(6):1243-1255.e7. doi:10.1016/j.molcel.2019.06.019
4. Schirle NT, Sheu-Gruttadauria J, MacRae IJ. Structural basis for microRNA targeting. *Science (80-)*. 2014;346(6209):608-613. doi:10.1126/science.1258040
5. Rüdel S, Flatley A, Weinmann L, Kremmer E, Meister G. A multifunctional human Argonaute2-specific monoclonal antibody. *RNA*. 2008;14(6):1244-1253. doi:10.1261/rna.973808
