# Supplementary material for: Single-molecule FRET uncovers hidden conformations and dynamics of human Argonaute 2
Source: Nat Commun. 2022 Jul 2;13:3825. doi: 10.1038/s41467-022-31480-4 (PMC9250533; doi:10.1038/s41467-022-31480-4)
Supplement: Supplementary file 3 — Reporting Summary [file 41467_2022_31480_MOESM3_ESM.pdf]

nature portfolio

Corresponding author(s): Dina Grohmann, Sarah Willkomm

Last updated by author(s): May 30, 2022

Reporting Summary

Nature Portfolio wishes to improve the reproducibility of the work that we publish. This form provides structure for consistency and transparency in reporting. For further information on Nature Portfolio policies, see our Editorial Policies and the Editorial Policy Checklist.

Statistics

For all statistical analyses, confirm that the following items are present in the figure legend, table legend, main text, or Methods section.

n/a	Confirmed	
☐	☒	The exact sample size (*n*) for each experimental group/condition, given as a discrete number and unit of measurement
☐	☒	A statement on whether measurements were taken from distinct samples or whether the same sample was measured repeatedly
☒	☐	The statistical test(s) used AND whether they are one- or two-sided<br>*Only common tests should be described solely by name; describe more complex techniques in the Methods section.*
☒	☐	A description of all covariates tested
☒	☐	A description of any assumptions or corrections, such as tests of normality and adjustment for multiple comparisons
☐	☒	A full description of the statistical parameters including central tendency (e.g. means) or other basic estimates (e.g. regression coefficient) AND variation (e.g. standard deviation) or associated estimates of uncertainty (e.g. confidence intervals)
☒	☐	For null hypothesis testing, the test statistic (e.g. *F*, *t*, *r*) with confidence intervals, effect sizes, degrees of freedom and *P* value noted<br>*Give P values as exact values whenever suitable.*
☒	☐	For Bayesian analysis, information on the choice of priors and Markov chain Monte Carlo settings
☒	☐	For hierarchical and complex designs, identification of the appropriate level for tests and full reporting of outcomes
☒	☐	Estimates of effect sizes (e.g. Cohen's *d*, Pearson's *r*), indicating how they were calculated

Our web collection on statistics for biologists contains articles on many of the points above.

Software and code

Policy information about availability of computer code

Data collection	Andor Solis Version 4.31, Andor Technology
Data analysis	iSMS- Single-Molecule FRET Microscopy Software Version 1.06 (including implemented vbFRET algorithm (Bronson et al., 2009, Biophysical journal, https://doi.org/10.1016/j.bpj.2009.09.031) , Origin lab 2019b

For manuscripts utilizing custom algorithms or software that are central to the research but not yet described in published literature, software must be made available to editors and reviewers. We strongly encourage code deposition in a community repository (e.g. GitHub). See the Nature Portfolio guidelines for submitting code & software for further information.

Data

Policy information about availability of data

All manuscripts must include a data availability statement. This statement should provide the following information, where applicable:

- Accession codes, unique identifiers, or web links for publicly available datasets
- A description of any restrictions on data availability
- For clinical datasets or third party data, please ensure that the statement adheres to our policy

The data underlying data generated in this study Figs. 1 – 5 and Supp. Figs. S3, S6 -S9 are available as a source data file, which is provided along. Source data are provided with this paper. PDB files of hAgo2 crystal structures that were used in this study are found using the following PDB identifiers: 4W5N, 4W5T, 6MDZ, 6NIT, 6N4O. Remaining raw datasets are available from the corresponding author upon reasonable request.

Field-specific reporting

Please select the one below that is the best fit for your research. If you are not sure, read the appropriate sections before making your selection.

☒ Life sciences ☐ Behavioural & social sciences ☐ Ecological, evolutionary & environmental sciences

For a reference copy of the document with all sections, see nature.com/documents/nr-reporting-summary-flat.pdf

Life sciences study design

All studies must disclose on these points even when the disclosure is negative.

Sample size	As appropriate for single-molecule FRET measurements, we carried out independent biological replicates for each experiment (see molecule numbers and number of replicates in Supplementary Table 2). Minimal sample sizes were chosen based on the sample variability and heterogeneity and were not pre-determined using any statistical method.
Data exclusions	We only excluded data that arose from measurements with technical problems with the TIRF setup. (1 measurement with hAgo2-PAZ and guide14Cy5, 1 measurement with hAgo2-PAZ/Mid-guide RNA-target RNA longBiotin, 1 measurement with hAgo2-PAZ-guide14Cy5-Biotinlongtarget mm9-12, and 1 measurement with hAgo2Mid-pas2bCy5, in these measurements technical problems with the setup occured, e.g. not fully illuminated field of view).
Replication	The number of replicates of all single molecule measurements can be found in Supplementary Table 2 and all replicates were biological replicates and led to highly reproducible data sets (compare standard deviations in Supplementary Table 2)
Randomization	Randomization of samples in this study was not necessary, because we did not allocate variants of hAgo2 proteins to different groups.
Blinding	Samples were not allocated into groups and hence, blinding was not required.

Reporting for specific materials, systems and methods

We require information from authors about some types of materials, experimental systems and methods used in many studies. Here, indicate whether each material, system or method listed is relevant to your study. If you are not sure if a list item applies to your research, read the appropriate section before selecting a response.

Materials & experimental systems

n/a	Involved in the study	
☐	☒	Antibodies
☐	☒	Eukaryotic cell lines
☒	☐	Palaeontology and archaeology
☒	☐	Animals and other organisms
☒	☐	Human research participants
☒	☐	Clinical data
☒	☐	Dual use research of concern

Methods

n/a	Involved in the study	
☒	☐	ChIP-seq
☒	☐	Flow cytometry
☒	☐	MRI-based neuroimaging

Antibodies

Antibodies used	Rb anti-Rt IgG (H+L) Secondary antibody, Biotin conjugate, Prod# 31834 Lot# SK2483271 Invitrogen; Rat anti-mouse IgG Biotin Secondary Antibody, Prod# 13-4013-85 Lot# E02828-1635 eBioscience; MsmAb to Argonaute-2, Prod# ab57113, Lot#GR285355-3 Lot# GR251959-1, abcam; 11A9 Rüdel et al., 2008, doi:10.1261/rna.973808; IRDye 800CW Goat anti-Rat IgG antibody (secondary antibody) Prod#926-32219 Lot#C90122-01, Li-Cor Biosciences; Goat anti-Rat IgG Alexa Fluor647 Secondary antibody, Prod# A21247, Lot# 2311802 Invitrogen
Validation	ab57113: "Our Abpromise guarantee covers the use of ab57113 in the following tested applications: WB, ..."<br>11A9: Rüdel et al., 2008; doi: 10.1261/rna.973808<br>31834: "Product # 31834 has been successfully used in Western blot, IF, ICC, IHC and ISH applications." Thermofisher<br>13-4013-85: "Applications Reported: In conjunction with enzyme- or fluorochrome-conjugated Streptavidin (SAV) or Avidin (AV), this polyclonal antibody can be used for detection of purified mouse immunoglobulins (IgG) in ELISA, immunoblotting (WB), immunohistochemical staining and flow cytometry. Applications Tested: This polyclonal antibody has been tested by flow cytometric analysis to detect purified mouse monoclonal antibodies." Thermo Fisher<br>926-32219: "This conjugate has been specifically tested and qualified for Western blot applications." licor biosciences<br>A21247: Antibody testing data for Westernblot available on the Thermo Fisher homepage.

Eukaryotic cell lines

Policy information about cell lines

Cell line source(s)	HEK293 T cells were supplied by the laboratory of Gunter Meister (University of Regensburg).
Authentication	We did not authenticate the cell line we used as hAgo2-production in these cell lines was successfull and we did not perform cell biological experiments that rely on the identity of the cell line (e.g. in protein co-purification studies).
Mycoplasma contamination	We did PCR to check for Mycoplasma contamination. The cells we used were negative in this test.
Commonly misidentified lines (See ICLAC register)	We did not use commonly misidentified cell lines.
